# Epidemiological Profiles of Foreign-Born and US-Born Hispanic Blood Donors in a Major Metropolitan Area in the United States

**DOI:** 10.1155/2012/820514

**Published:** 2012-02-06

**Authors:** Adelbert B. James, Cassandra D. Josephson, Marta I. Castillejo, George B. Schreiber, John D. Roback

**Affiliations:** ^1^Department of Pathology and Laboratory Medicine, Center for Transfusion and Cellular Therapies, Emory University School of Medicine, Atlanta, GA 30322, USA; ^2^Independent Consultant, Bethesda, MD 20814, USA

## Abstract

*Background.* The explosive growth of Hispanics in the US makes this population a significant and untapped source for blood donation. *Methods.* A cross-sectional study was performed to evaluate blood donation behaviors and demographics of foreign-born and US-born Hispanic donors between 2006 and 2009 in metropolitan Atlanta, GA, USA. Bivariate analyses and multivariate logistic regression were used to assess factors associated with foreign-born donors. *Results.* 5,119 foreign-born and 11,841 US-born Hispanics donated blood. Foreign-born Hispanic donors were more likely than US-born donors to be blood group O (57.6% versus 52.0%; *P* < .001) and more frequent donors (2.2 versus 2.0; *P* < .001). Cuban-born donors had the highest rates of return donation (63.2%). In contrast, Mexicans, the most prevalent subpopulation among foreign-born Hispanic donors (31.8%), had the lowest rates of return donation (42.0%). *Conclusions.* The heterogeneity found among Hispanic donors in this study is valuable for the design of recruitment strategies to increase blood donations.

## 1. Introduction

In 2010, Hispanics accounted for more than half of the growth in the total US population between 2000 and 2010, making them the fastest growing ethnic group [1]. This expansion of Hispanics is largely due to the natural increase (births minus deaths) of the existing population. Since 2000, of the total Hispanic growth in the US, 40% of the increase was due to net international migration [2]. In numbers, Hispanics currently exceed African Americans and account for 15.1% of the total US population [2]. Particularly, the South has experienced a larger overall Hispanic growth than any other region in the US [2]. In Georgia, the Hispanic population increased 96.1% from 453,227 in 2000 to 853,689 in 2010; the overall total population increase between 2000 and 2010 was 18.3% [3].

This explosive growth of Hispanics in the US makes these residents a vital and underutilized source of potential blood donors. Currently, in Georgia, Hispanics donate at significantly lower rates than non-Hispanic whites and African Americans [4]. Approximately 1.4% of Hispanics donate blood each year when compared to 4.2% of non-Hispanic whites and 2.4% of African Americans [4]. The disparity between these groups' donation frequencies is especially vexing since a high percentage of Hispanics (57–70%) are blood group O [5] and their inclusion in the donor pool could significantly improve blood supply logistics.

In an effort to increase blood donation among Hispanics, an evaluation of their culture, education, language obstacles, and knowledge of blood donation is essential to understanding the observed donation differences. “Hispanics,” however, are not a single monolithic culture. In the US, there are five primary Hispanic subgroups—Cuban, Central and South American, Mexican, and Puerto Rican [6, 7]. These subgroups are based upon geographic distributions of Hispanic populations within the US. For example, the majority of Cubans reside in Florida; Central Americans and South Americans reside mostly in the south, northeast, and the west; Mexicans reside primarily in the Southwest and the majority of Puerto Ricans live in the Northeast of the US [7]. Cultural differences exist between the primary Hispanic subgroups [8], and additional divergences exist with blending of Hispanics from distinct subgroups. 

Acculturation is another important factor influencing Hispanics differently depending on whether one is born in, rather than immigrates to, the US. To this end, there is a need for more epidemiologic-based blood donor studies that focus on Hispanic subgroups.

Knowledge of the demographics of Hispanic donors could lead to a better understanding of Hispanic blood donation behaviors and is an important step for developing effective recruitment strategies that may help increase blood donations. Thus, the purpose of this study is to evaluate the demographics and patterns of blood donation of Hispanics, foreign-born, and US-born.

## 2. Materials and Methods

### 2.1. Study Population and Design

Data from the Retrovirus Epidemiology Donor Study-II (REDS-II) database at the American Red Cross Blood Services (ARC), Southern Region (Atlanta, GA), were included in this study. Donors were selected for this study based on their self-identification as being Hispanic from 19 Spanish-speaking countries and the US. The selection criteria also included ages between 16 and 82 years who gave between one and twenty-four whole blood donations from 2006 to 2009 at the ARC in metro Atlanta. Donors selected their country of birth from a list that included South American, Central American, and Caribbean countries. Since the US was included in the list to choose from, Hispanic donors could be categorized as either foreign-born or US-born. For purposes of this analysis, foreign-born donors were classified as Cuban, Central American, South American, Mexican, and Puerto Rican. (Donors from the Dominican Republic were classified as Central Americans.)

### 2.2. Statistical Analyses

Study variables consisted of date of donation, age, gender, highest level of education attained, history of deferral, history of blood transfusion, site of donations (fixed or mobile centers), and ever pregnant. This was a cross-sectional study using bivariate analyses for variables of interest. The mean donation frequency was calculated by dividing the total number of donations by the total number of blood donors. Donor rates were calculated as the total number of successful donations in counties where blood was collected over the total Hispanic subgroup population living in Georgia using data from the 2006–2008 US Census. Deferral rates were calculated by the number of deferrals divided by the total number of deferrals and successful presentations and expressed per 1,000 donor presentations. For this analysis, screening test positives rather than confirmatory testing results were used to calculate the proportion of deferrals. We used odds ratios (ORs), 95% confidence intervals (CI), and chi-square analyses to examine the relationships between foreign- versus US-born donors and donation sites. In addition, multivariate logistic regression model was constructed to predict whether the donor was foreign-born versus US-born using gender, age, education, first-time donor status, history of transfusion and deferrals, and donation site as independent variables. Data were analyzed by SPSS statistics 19 (SPSS Inc., 2009, Chicago, IL).

## 3. Results

The study population consisted of 5,119 foreign-born (30.2%) and 11,839 US-born Hispanic blood donors (69.8%) between 2006 and 2009 (see Table 1). Foreign-born Hispanics represented 0.7% Cuban, 7% Central American 7.9% South American, 9.6% Mexican, and 4.9% Puerto Rican of all Hispanic donors. More than half of all donors were females (56.2%), and donor ages ranged between 16 and 82 years with 73.7% of donors younger than 35 years. Overall, the mean age was 27.3 ± 12.4 years. The mean age among foreign-born donors was higher (30.8 ± 13.2 years) than US-born donors (25.8 ± 11.7 years) (*P* < .001).

Educational levels varied among subgroups. A greater percent of donors from Cuba (55.9%) and Puerto Rico (50.2%) represented the highest educated donors followed by South Americans (46.4%), US-born (35.0%), Central Americans (27.0%), and Mexican donors (14.3%). Mexican donors represented the highest percent of donors without a high school diploma (22.1%) followed by Central Americans (9.5%), US-born (3.8%), South Americans (3.2%), Cubans (2.9%), and Puerto Ricans (2.1%). The majority of donors with less than a 9th grade education were Mexicans (13.5%) and Central Americans (6.5%). There was no significant difference in earning a high school diploma or bachelor's degree between foreign-born and US-born donors. 

The numbers of screening test positive donors for cytomegalovirus (CMV) (834), hepatitis B (104), syphilis (58), hepatitis C (HCV) (35), human immunodeficiency virus (HIV) (25), and Chagas disease (7) were relatively low. The majority of CMV (459) and HIV (23) infections were diagnosed among US-born Hispanics. Most HCV infections were diagnosed among US-born Hispanics (23), Puerto Ricans (5), and Mexicans (4). Overall, US-born Hispanics were more likely to be infected with HIV than foreign-born Hispanics (0.2% versus 0.0%, *P* = .015); less likely to be infected with hepatitis B (0.5% versus 1.0%, *P* = .001); less likely to be infected with syphilis (0.3% versus 0.6%, *P* = .005); less likely to be infected with CMV (49.9% versus 70.8%, *P* < .001); less likely to be infected with Chagas disease (0.0% versus 0.2%, *P* = .002). There was no statistically significant difference in HCV infection between US-born Hispanics and foreign-born Hispanics. 

**Figure 1:**
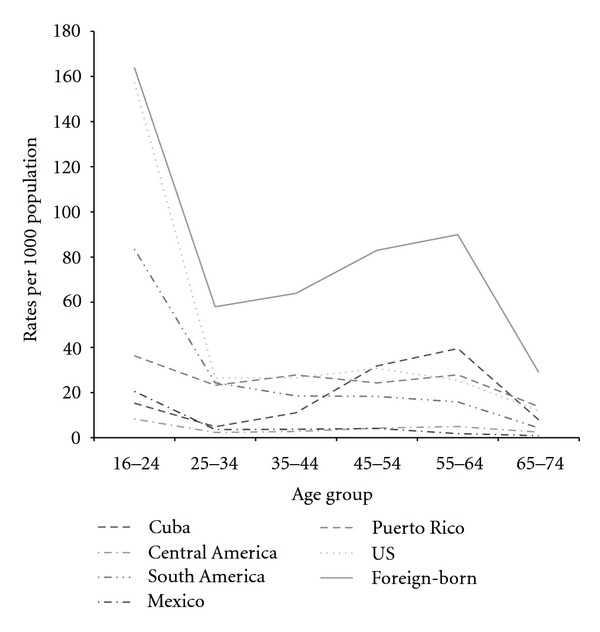
Blood donor rates among Hispanic subgroups in Georgia per 1,000 population.

Figure 1 illustrates the rate of donor representation by subgroup in Georgia. Overall, the age-relevant donor rate including both foreign-born and US-born Hispanics was 34 per 1,000 population. Donor rates were sharply diminished among donors after the age of 24 years and were considerably varied across age groups. The rate among US-born donors (55 per 1,000 population) was higher when compared to foreign-born donors (18 per 1,000 population). The donor rate among younger Mexicans (16–24 yrs old) decreased from 21 per 1,000 population compared to a rate of 2 per 1,000 population among older Mexicans (55–64 yrs old). The donor rates of Cuban, South American, and Puerto Rican donors were consistently higher than the rates of Central American and Mexican donors. 

 Overall, donor deferral rates ranged from 123 per 1,000 presentations among Puerto Ricans to 177 per 1,000 presentations among South Americans. The deferral rate among US-born Hispanics was 136 per 1,000 presentations. The most common reasons for deferral were low hematocrit (Hct) or low hemoglobin (Hb), malaria travel, feeling unwell, and high blood pressure or pulse. South Americans (85 per 1,000 presentations), Cubans (74 per 1,000 presentations), and Central Americans (68 per 1,000 presentations) had the highest deferral rates due to low Hct or Hb; the deferral rate among US-born Hispanics was 60 per 1,000 presentations. Central Americans (17 per 1,000 presentations) and South Americans (10 per 1,000 presentations) had the highest deferral rates due to travel to a malaria endemic area; the deferral rate among US-born Hispanics was 6 per 1,000 presentations. 

### 3.1. Hispanic Donors Born in the US

Women were the predominant US-born Hispanic donors (57.3%) of whom the majority were between 16 and 34 years old (62.0%). The prevalence of O type blood was 52.0%. At least 35.0% earned a bachelor's degree, and 17.4% received a high school diploma. Approximately 48.8% were first-time donors; 58.8% donated blood once; 10.1% had a history of deferrals; 9.1% donated at a fixed site. Overall, US-born donors were less likely to return than foreign-born donors (41.2% versus 46.5%, *P* < .001); 2 donations (20.6% versus 24.4%, *P* < .001); 3 donations (11.1% versus 14.8%, *P* < .001); 4 donations (6.7% versus 9.0%, *P* < .001); 5 donations, (4.7% versus 6.3%, *P* < .001); 6 donations (3.2% versus 4.4, *P* < .001); ≥7 donation. 

### 3.2. Foreign-Born Donors

A preponderance of foreign-born Hispanic donors were female (53.4%), mostly between the ages of 16–34, (63.3%). Foreign-born donors were more likely to be male than US-born donors (OR = 1.13, 95% CI: 1.09–1.29) and less likely to be younger (16–34 years) than donors born in the US (OR = 0.48, 95% CI: 0.45–0.66). At least 34.1% earned a Bachelor's degree and 17.7% had received a high school diploma. As a group, foreign-born Hispanics were more likely to be blood type O and become repeat donors; 57.6% of foreign-born donors were blood type O, and these donors were 24% more likely to be repeat donors than US-born donors (OR = 1.24, 95% CI: 1.16–1.32). Foreign-born donors were more likely to have a history of deferrals than US-born donors (OR = 1.18, 95% CI: 1.06–1.31). 

The logistic regression model showed predictors for foreign-born donors. After adjustment of variables in the model, foreign-born donors were 20% more likely to use fixed donation centers than US-born donors (OR = 1.20; 95% CI: 1.06, 1.35). Foreign-born donors were 17% more likely to be males than US-born donors (OR = 1.17, 95% CI: 1.09, 1.27); foreign-born donors were more likely to be first-time donors than US-born donors (OR = 1.30, 95% CI: 1.18, 1.42) (see Table 2). 

Table 3 shows that foreign-born donor characteristics were not homogenous but rather varied with the country of birth. The highest percentage of group O donors were found among Mexicans (63%), followed by South American donors (57.2%), Central American donors (57%), Puerto Rican donors (50.3%), and Cuban donors (45.4%). Cuban-born Hispanics also had the highest rates of return donation; the percent of repeat donors between donating between 2 and at least 7 donations was 63.2%, 43.2%, 30.4, 22.4%, 15.2%, and 9.6%, respectively. The frequencies of repeat donations per year among the other foreign-born Hispanics are South American (51.6%, 27.3%, 17.1%, 10.8%, 7.8%, and 5.7%), Puerto Rican (49.2%, 29.6%, 18.8%, 11.4%, 8.6%, and 6.4%), Central American (43.4%, 24.1%, 16.0%, 10.0%, 7.3%, and 4.6%), and Mexican (42.0%, 18.0%, 9.0%, 5.0%, 3.0%, and 1.8%). Mexican donors had the lowest percentage of historical deferrals (9.3%), followed by Puerto Ricans (10.3%), Central Americans (12.2%), South Americans (14.6%), and Cubans (16%). 

### 3.3. Mean Donation Frequency

The mean donation frequency (2.0 donations per year) varied by demographic group and ranged from 1.8 among Mexicans to 3.1 among Cubans. The mean donation frequency of foreign-born Hispanic donors was higher (2.2) when compared to US-born donors (2.0. *P* < .001). Donors who visited fixed donor centers had a higher mean donation frequency (3.1) compared to donors who visited mobile sites (1.0, *P* < .001). Donors who were older (35–82 years) had a higher mean donation frequency (2.6) compared to younger donors (1.8) (*P* < .001). During this study period, 603 repeat donors contributed, at a minimum, 7 donations per year. The highest frequency of donations (24) was offered by one donor. Of the donors evaluated, 9701 (57.2%) donated one time, while 7257 (42.8%) donated two times (median return time 6.2 months). Of the remaining donors, 21.7% donated 3 times, 12.2% donated 4 times, 7.4% donated 5 times, 5.2% donated 6 times, and 3.6% donated blood at least 7 times.

## 4. Discussion

The findings in this study reveal variations in blood donation patterns among Hispanic groups in the US. The diversity in demographics between Hispanics who are foreign-born and Hispanics born in the US may account for these differences in donation practices. Foreign-born donors were more likely to be male and older compared to US-born donors (*P* < .001). Hispanics born abroad tend to be older and are therefore less likely to donate at school blood drives, the most common site for recruiting first-time donors. Hispanics born in the US tend to donate at a younger age and are more likely to participate as first time donors in school drives. 

Further analysis suggests that foreign-born donors were more likely to donate and become repeat donors than those born in the US. Some have suggested that Hispanic immigrants are not a random sample of their home countries since they tend to have more resources, education, psychological strength, and motivation to emigrate [9–11], and may be more willing to donate blood. Other researchers have shown that age, gender, and education are strong predictors for blood donations [4]. The findings of this study show that the Hispanic donor population is more highly educated than the US Hispanic population [12]. This study echoes previous findings; the donor rates of non-Hispanic whites (68.9/1,000 population) and African Americans (34.9/1,000 population) are significantly higher than that of Hispanics (34.1/1,000 population) [4]. 

This study demonstrates that Mexicans were least likely to donate blood when compared to all Hispanic subgroups, including those born in the US. This is troubling since Mexicans are the largest subgroup, representing 63% of the total Hispanic population living in the US in 2010 [1, 3, 13]. An estimated 32 million Mexicans account for three-fourths of the 15.2 million increase in the total Hispanic population between 2000 and 2010 [1], including more than half who represent undocumented immigrants [9]. The results of this current study, revealing the highest prevalence of Universal blood type O among Mexicans (63%) and the lowest rates of previous deferrals, emphasize the need for recruitment strategies targeted at this subgroup in an effort to expand the current blood supply. 

Enrollment programs must necessarily address the immigration concerns in the wake of recently enacted immigration laws and the reluctance perhaps to show a photo ID needed to donate blood. The findings in this and other studies of the relatively low level of education among Mexicans [14] also support enlistment strategies that provide blood donor education to this subgroup, at least 22% of whom have not graduated from high school. Possible donor selection bias towards Hispanics should be evaluated that might be contributing to underrepresentation of Hispanics in the donor base, namely, of Mexicans. Factors such as lower education and economic status might be significant barriers to donation. To this end, mobile drive campaigns could target sites or areas that appear to represent potential recruiting grounds for prospective Hispanic donors. In addition, enlistment of all Hispanics should be offered in their native Spanish for both newly immigrants and older generations of potential donors to increase their degree of comfort and understanding of donating blood. 

Among the Hispanic subgroups, Colombians, Cubans, and Puerto Ricans were more likely to, and consistently, donate (*P* < .001). For example, Cubans were among the highest to repeat with a second donation (63%) and donated most frequently (3.1). The high rates of Cuban blood donors may be due to the efficiency of Cuba's national blood system that may mandate blood donations. For instance, Cuba has the highest blood donation rate in Latin America and the Caribbean (439.6/10,000 inhabitants) [15]. The donation rates in Colombia and Mexico are 115.7/10,000 and 126.2/10,000 inhabitants, respectively [15]. 

The World Health Organization (WHO) and the International Federation of the Red Cross recognize that a country must collect the equivalent of 3%–5% of its population to ensure an adequate volunteer blood supply. In comparison, the US and Latin America collect blood from just slightly over 4.0% and 1.4% of their population, respectively [16]. Complicating recruitment planning, this analysis demonstrated that Cuban donors had the highest percentage of deferrals, with up to 50% of perspective donors having a low hemoglobin level—yet another fact to consider in donation planning strategies. 

In another study finding, foreign-born donors were more likely to visit a fixed site than a mobile site, and return for repeat donations much earlier is probably due to the impact of recruitment efforts. Individuals who want to donate more frequently often make return visits to fixed sites [17, 18]. It can be speculated that higher donor rates occur at fixed sites because of the center's availability, whereas donors at a mobile must depend on the frequency and convenience of the mobile rather than the donor's. Sites such as workplaces might frequently underrepresent the Hispanics makeup of the areas. Thus, policy makers could consider areas of heavily populated Hispanics and perhaps survey potential donors in these areas to consider the most convenient times for the blood mobiles to visit these communities. Education on the importance and ease of blood donation could target the areas to stimulate reasons to donate. 

The need to emphasize the need for and merit of altruistic blood donations is important in the Hispanic community. To approximate the rate of blood donation of non-Hispanic whites in the US, Hispanics would have to triple their blood donors each year [5]. It is known that there is a greater willingness among Hispanics to offer blood for family members [19] yet limit donations for the wider community as a consequence of a dearth of appropriate donor recruitment and a lack of knowledge and/or mistrust of the US blood system [20]. Since directed donation is deemphasized in the US, the need for family donations is limited. Fortunately, the last decade has seen a shift to promote voluntary donations in Latin America and the Caribbean, where blood safety can be assured. The proportion of voluntary blood donors in Latin America and the Caribbean increased from 15% in 2001 to 36% in 2003 [15, 21]. 

This study provides substantial insight into the complexities and diversities among Hispanic donors. While donors from this study are from a limited metropolitan area and might not represent Hispanic populations in other areas of the country, it demonstrates that donors from numerous countries have cultural, social, economic, and educational divergences that may have an impact on their blood donations in the US. While current epidemiologic data are not collected solely on cultural subgroups, studies could be proposed in predominantly Puerto Rican, Colombian, or Cuban communities that could help highlight and report their divergences from another subgroup with regards to their knowledge of blood donation. The limitations of this study consist in the small number of foreign-born donors to analyze per country, such as Cuba. The study was also unable to determine the number of years lived in the US by foreign-born donors that may have impacted their blood donation knowledge and practices. Additionally, distinct acculturation processes over time and among subgroups of Hispanics may have profoundly influenced donation practices. 

A study of Hispanic donors, powered to reach a greater number of Hispanics and yield greater distinctions among the individual and subgroup demographics and cultural patterns, is needed. Blood donation recruiters are the yeomen of the blood supply chain who rely on information describing current Hispanic blood donation practices to guide them in the formulation of new recruitment strategies and programs. The growing size and importance of the Hispanic Community efforts, including education adapted to Hispanic subgroups, could render a larger and more diverse donor pool which offers the “priceless” gift of life.

##  Funding

1 P01 HL 086773: Mechanisms and interventions addressing serious hazards of transfusion and cellular therapies.

##  Conflict of Interests

The authors declare that there is no conflict of interests.

## Figures and Tables

**Table 1:** Characteristics of US-born and foreign-born Hispanic donors.

	US-born		Foreign-born		
	Number	%	Number	%	*P* value

All donors	11,839	69.8	5,119	30.2	
Gender					<.001
Male	5,053	42.7	2,383	46.5	
Female	6,786	57.3	2,738	53.5	
Age groups (years)*					<.001
16–24	7,324	61.9	2,125	41.6	
25–34	1,925	16.3	1,117	21.9	
35–44	1,447	12.2	973	19.0	
45–54	789	6.7	606	11.9	
55–64	281	2.4	239	4.7	
65–82	64	0.5	52	1.0	
Mean age	25.8		30.8		<.001
Education*					<.001
9–12th grade, no diploma	4,566	42.2	1,516	34.3	
High-school graduate	1,526	14.1	686	15.5	
Some college or tech	2,768	25.6	1,136	25.7	
Bachelor's degree	1,950	18.0	1,079	24.4	
Mean donation frequency	2.0		2.2		<.001
First-time donor?*					<.001
Yes	5,389	48.8	2,148	45.7	
No	5,658	51.2	2,552	54.3	
History of blood transfusion?*					0.074
Yes	246	2.3	123	2.7	
No	10,516	97.7	4,466	97.3	
History of deferral?					.001
Yes	1,190	10.1	599	11.7	
No	10,649	89.9	4,520	88.3	
Donated at fixed sites?*					<.001
Yes	1,074	9.1	612	12.0	
No	10,763	90.9	4,506	88.0	
Infectious disease					
CMV	459	3.9	375	7.3	<.001
Hepatitis B	56	0.5	48	0.9	.001
Hepatitis C	23	0.2	12	0.2	.713
Syphilis	30	0.3	28	0.6	.005
HIV	23	.2	2	0.0	.015
Chagas disease	1	0.0	6	0.2	.002

*Missing data.

**Table 2:** Logistic regression model examining factors associated with foreign-born Hispanic blood donors.

	Unadjusted OR (95% CI)	*P* value	Adjusted OR (95% CI)	*P* value
Gender				
Male	1.17 (1.09, 1.25)	<.001	1.17 (1.09, 1.27)	<.001
Females	1.0		1.0	
Age groups (years)				
16–24	1.0		1.0	
25–34	2.00 (1.83, 2.18)	<.001	2.47 (2.19, 2.78)	<.001
35–44	2.32 (2.11, 2.54)	<.001	2.85 (2.50, 3.25)	<.001
45–54	2.65 (2.36, 2.97)	<.001	3.46 (2.97, 4.03)	<.001
55–64	2.93 (2.45, 3.51)	<.001	4.11 (3.30, 5.10)	<.001
65–82	2.80 (1.94, 4.05)	<.001	3.61 (2.35, 5.54)	<.001
Education				
9–12th grade, no diploma	1.0		1.0	
High school graduate	1.35 (1.22, 1.51)	<.001	0.94 (0.83, 1.07)	.359
Some college or tech	1.24 (1.13, 1.35)	<.001	0.75 (0.67, 0.85)	<.001
Bachelor degree	1.67 (1.52, 1.83)	<.001	0.77 (0.67, 0.89)	<.001
First-time donor?				
Yes	0.88 (0.82, 0.95)	<.001	1.30 (1.18, 1.42)	<.001
No	1.0		1.0	
History of transfusion?				
Yes	1.18 (0.95, 1.47)	.134	0.88 (0.69, 1.11)	.276
No	1.0		1.0	
History of deferral?				
Yes	1.19 (1.07, 1.32)	.001	1.09 (0.96, 1.23)	.183
No	1.0		1.0	
Donation site				
Fixed site	1.36 (1.22, 1.51)	<.001	1.20 (1.06, 1.35)	.005
Mobile site	1.0		1.0	

**Table 3:** Characteristics of foreign-born and US-born Hispanic donors by country/region of interest.

	Cuba		Central America		South America		Mexico		Puerto Rico		US-born	
	Number	%	Number	%	Number	%	Number	%	Number	%	Number	%

All donors	125	0.7	1,185	7.0	1,346	7.9	1,629	9.6	834	4.9	11,839	69.8
Gender												
Male	63	50.4	533	45.0	635	47.2	773	47.5	379	45.4	5,053	42.7
Female	62	49.6	652	55.0	711	52.8	856	52.5	455	54.6	6,786	57.3
Age groups (years)												
16–24	16	13.0	450	38.0	501	37.2	915	56.2	243	29.2	7,324	61.9
25–34	7	5.7	291	24.6	277	20.6	354	21.8	188	22.6	1,925	16.3
35–44	27	21.9	235	19.8	269	20.0	246	15.1	196	23.6	1,447	12.2
45–54	46	37.4	134	11.3	210	15.6	93	5.7	123	14.8	789	6.7
55–64	22	17.9	62	5.2	77	5.7	16	1.0	62	7.5	281	2.4
65–82	5	4.1	13	1.1	12	0.9	3	0.2	19	2.3	64	0.5
Mean age	45.0	—	31.4	—	32.3	—	26.1	—	35	—	25.8	—
M. donation freq.	3.1		2.1		2.4		1.8		2.4		2.0	
Hx. of Transfusion	6	4.8	29	0.0	33	2.4	31	1.9	24	2.9	246	2.1
Hx. of deferral	20	16.0	145	12.2	197	14.6	151	9.3	86	10.3	1,190	10.1
Donated at fixed	18	14.4	146	12.3	227	16.9	126	7.7	95	11.4	1,074	9.1
Ever pregnant	42	73.7	321	52.4	308	47.2	320	39.5	233	54.4	2,292	35.8
Infectious disease												
CMV	7	5.6	88	7.4	146	10.8	96	5.9	38	4.6	459	3.9
Hepatitis B	1	0.8	19	1.6	6	0.4	15	0.9	7	0.8	56	0.5
Hepatitis C	0	0	1	0.1	2	0.1	4	0.2	5	0.6	23	0.2
Syphilis	0	0	11	1.0	7	0.5	9	0.6	1	0.1	30	0.3
HIV	0	0	1	0.1	0	0	1	0.1	0	0	23	0.2
Chagas disease	0	0	0	0	1	0.1	5	0.3	0	0	1	0.0

M.: mean; Hx.: history.
